# Plasma histone monomers as novel diagnostic markers in adult glioblastoma

**DOI:** 10.1016/j.jlb.2026.100464

**Published:** 2026-04-05

**Authors:** Desislava K. Tsoneva, Diana Buzova, Stefani Mariyanova Todorova, Yavor Enchev, Jan Frohlich, George N. Chaldakov, Anton B. Tonchev, Jan Cerveny, Manlio Vinciguerra

**Affiliations:** a Department of Medical Genetics, Medical University of Varna, Varna, Bulgaria; b Department of Stem Cell Biology and Transplantology, Research Institute of the Medical University of Varna, Varna, Bulgaria; c Department of Adaptive Biotechnologies, Global Change Research Institute CAS, Brno, Czech Republic; d Department of Neurosurgery and ENT, Medical University Varna, Varna, Bulgaria; e Clinic of Neurosurgery, University Hospital "St. Marina", Varna, Bulgaria; f International Clinical Research Center, St Anne's University Hospital, Brno, Czech Republic; g Department of Anatomy and Cell Biology, Research Institute of the Medical University of Varna, Varna, Bulgaria; h LUM University, Casamassima, Italy

**Keywords:** Glioblastoma, histones, Imaging flow cytometry, Epigenetics, Liquid biopsy

## Abstract

**Background:**

Glioblastoma (GB), the most aggressive primary brain tumor in adults, has a median survival of 12-18 months and lacks reliable minimally invasive biomarkers. Traditional diagnostics are limited by invasiveness and the inability to capture dynamic tumor changes. Circulating histones, reflecting tumor burden and epigenetic alterations, have emerged as potential liquid biopsy markers, but studies in adult GB are scarce compared to pediatric gliomas. The aim of this study was to characterize the plasma histone profile in adult GB patients versus healthy controls and evaluate its diagnostic potential.

**Methods:**

Plasma samples from 44 adult GB patients and 30 age-matched healthy controls were analyzed. Plasma levels of histones H3.1 and H3.3 were quantified by ELISA. Individual histones (H2A, H2B, H3, H4, macroH2A1.1, macroH2A1.2) and histone complexes (dimers/tetramers) were measured using an established imaging flow cytometry (ImageStreamX) multichannel detection approach.

**Results:**

GB patients exhibited significantly elevated plasma levels of individual histone monomers H2A, H3, macroH2A1.1, and macroH2A1.2, as well as markedly reduced H4 levels, compared to controls. Levels of histones H3.1- and H3.3 detected in plasma, showed no significant differences. MacroH2A1.2 levels negatively correlated with age in males.

**Conclusions:**

Adult GB displays a distinct circulating histone signature dominated by elevated free monomers rather than nucleosome complexes, contrasting with pediatric gliomas. These findings provide proof of concept for plasma histone monomers as novel minimally invasive diagnostic biomarkers in adult glioblastoma.

**Key points**

● Adult glioblastoma patients show elevated plasma levels of free histone monomers (H2A, H3, macroH2A1.1, macroH2A1.2) and reduced H4 vs controls.
● Plasma levels of nucleosome complexes were unchanged, suggesting monomer dysregulation as a key signature.
● Circulating histone profiles in adult glioblastoma are distinct from pediatric gliomas, indicating age-specific differences.

**Importance of the study**

This study addresses a critical gap in glioblastoma (GB) diagnostics by characterizing circulating histone profiles in adult patients, an area previously underexplored compared to pediatric gliomas. Using advanced imaging flow cytometry and ELISA, we identify a distinct plasma signature in adult GB: significantly elevated free histone monomers (H2A, H3, macroH2A1.1, macroH2A1.2) with reduced H4, while nucleosome complexes remain unchanged. These results contrast sharply with pediatric H3K27-altered gliomas, where complexes predominate. These findings highlight age-specific differences in histone release mechanisms and provide proof of concept for free histone monomers as novel, minimally invasive liquid biopsy biomarkers for adult GB detection and potential grading. By linking monomer elevation to hematopoietic/immune contributions rather than solely tumor necrosis, the study opens avenues for non-invasive epigenetic monitoring, improved early diagnosis, and deeper insight into GB-driven systemic immunosuppression, paving the way for larger validation studies and clinical translation.

## 1 Introduction

Glioblastoma (GB), classified as a World Health Organization (WHO) grade IV tumor, is the most prevalent, aggressive, and lethal primary brain tumors in adults, presenting significant challenges in diagnosis and management due to its infiltrative nature and molecular heterogeneity [1]. GB has a median survival time of around 12-18 months after diagnosis [2]. Traditional diagnostic approaches, such as imaging and tissue biopsy, are often limited by invasiveness, sampling bias, and the inability to capture dynamic changes in tumor biology. The emergence of liquid biopsy strategies, particularly the detection of circulating tumor-derived molecules, offers a promising avenue for minimally invasive GB diagnosis and monitoring [3]. Among these, histones and their modifications have garnered attention as potential biomarkers and epigenetic drivers of GB, reflecting both tumor burden and molecular characteristics [4]. Histones are highly conserved, positively charged proteins that play a central role in the organization and regulation of eukaryotic chromatin [5]. The core histones H2A, H2B, H3, and H4 assemble into an octamer complex, around which DNA is wrapped to form the nucleosome, the fundamental repeating unit of chromatin [6]. Each core histone contains a conserved “histone fold” domain, which mediates dimerization and nucleosome assembly, and a flexible N-terminal “tail” that is subject to extensive post-translational modifications [7]. Linker histones, such as H1, bind to the nucleosome and regulate higher-order chromatin structure, possessing a structured globular domain and flexible termini [8].

Histone variants exist for most core histones except for H4, further diversifying nucleosome composition and chromatin function, which underpins the complexity of chromatin regulation and has direct implications for tumor biology [9]. In the context of GB, the molecular landscape of circulating histones is shaped by both canonical and variant forms. Notably, evolutionary conserved histone H3 subtypes, specifically H3.3 and H3.1, with ∼5 amino acids difference, are particularly relevant in pediatric high-grade gliomas. Recurrent mutations in the genes encoding histone variants H3F3A (H3.3) and HIST1H3B (H3.1) are defining features of several malignant pediatric brain tumors, including diffuse intrinsic pontine gliomas (DIPG) and diffuse midline gliomas (DMG) [10,11]. The most clinically significant mutations are missense changes in the histone H3 tail at lysine 27 (K27M), predominantly found in midline brain tumors and glycine 34 (G34 R/V), which are more common in hemispheric gliomas [11,12]. These mutations are not exclusive to pediatric brain tumors and have been identified in other tumor types, such as chondroblastoma and giant cell tumors of bone, and less frequently in adult brain tumors [13]. The discovery of these histone subtype mutations has led to the reclassification of certain brain tumors and provided new insights into their oncogenic mechanisms, tumor specificity, and potential for targeted therapies [14]. Independent of the mutations they may carry, H3.1 and H3.3 subtypes differ in their deposition timing, chaperones (CAF-1 versus HIRA, respectively), and genomic localization, with H3.1 being replication-coupled and marking silent regions, while H3.3 is replication-independent and deposited throughout the cell cycle, associated with active transcription and epigenetic maintenance and memory [15]. The release of histones into circulation in GB might be primarily associated with cell death and immune cell activation. Tumor cells undergoing necrosis or apoptosis release nuclear contents, including histones, into the extracellular space, from where they can enter the bloodstream or cerebrospinal fluid (CSF). Inflammatory processes and immune responses within the tumor microenvironment can also promote histone release. While direct studies on histone release mechanisms in GB are limited, the general paradigm in solid tumors suggests that both passive release from dying cells and active secretion by immune cells contribute to the pool of circulating histones [16]. The presence of circulating histones in the CSF and plasma of brain tumor patients reflects both tumor biology and the host response. Our recent advances in imaging flow cytometry, such as the ImageStream(X) platform, have enabled the detection and quantification of individual histones and histone complexes in the CSF of children with brain tumors, including GB [17]. This technology combines the quantitative power of flow cytometry with high-content image analysis, allowing for the identification of histones such as H2A, macroH2A1.1, macroH2A1.2, H2B, H3, H4, and H3 with the K27M mutation, as well as histone complexes [17]. In pilot studies, strong signals for individual histones and complexes were observed in the CSF of children with brain tumors, with H2A, macroH2A1.1, macroH2A1.2, and H3K27M being more abundant compared to other histones and complexes [17]. Among pediatric GB, diffuse midline glioma, H3 K27-altered (DMG) is a fatal tumor that arises in the midline structures of the brain. When located in the pons, it is more commonly referred to as diffuse intrinsic pontine glioma (DIPG). Recently, we reported a histone-based approach to diagnose DMG/DIPG in the plasma of pediatric patients. We found a significant increase in circulating histone dimers and tetramers (macroH2A1.1/H2B; macroH2A1.2/H2B; H2A/H2B; H3/H4; H2A/H2B/H3/H4) and a significant downregulation of individual histones (H2B; H4) [18]. Histones were detectable in the CSF of patients with DMG/DIPG and in the supernatant of DMG/DIPG cell lines, with patterns mostly similar to each other, but distinct compared to blood plasma [18]. Pediatric and adult GBs exhibit significant differences in their molecular biology (i.e. PDGFRA and H3K27M *versus* IDH mutations), localization within the brain (i.e. various *versus* cerebral hemisphere), rarity (rarer in children), prognosis, and metabolism (pediatric GB show higher glucose levels and reduced lactate compared to adult GB). In this respect, studies of circulating histone profiles in adult GB *versus* healthy adult subjects are lacking. The aim of this study was to address this gap. Our current findings provide proof of concept for histone-based liquid biopsies, which could enable minimally invasive epigenetic characterization of adult GB and facilitate diagnosis and prognosis.

## 2 Materials and methods

### 2.1 Patients and biofluids

The collection of human specimens was approved by the local Ethics Committee of the Medical University of Varna. All participants provided written informed consent before their enrolment in the study. We followed the standard and robust blood sampling/storage standard operation procedure (SOP) of the UK BioBank [19]. Blood samples were collected at the time of diagnosis before initiation of any specific treatment. Blood was collected in K2 EDTA-coated collection tubes and centrifuged at 3,000g for 20 min at 4 °C. The plasma samples were stored at −80 °C. A total of 74 patients were included in the study, of which 4 were recruited by the Department of Neurosurgery at the University Hospital “St. Marina” in Varna, Bulgaria. Neurological examination, imaging, and tissue biopsy were used to confirm the diagnosis, localization, and tissue of origin. 40 plasma samples from glioblastoma patients were acquired from Audobon Bioscience (TX, USA). Control samples (n = 30) were obtained from Proteogenex (CA, USA). All control samples were confirmed negative for HIV, HBV, HCV, and syphilis. All acquired samples were collected in K2 EDTA-coated collection tubes. All samples used in the study are from patients of Caucasian ethnicity. The following parameters were recorded: sex, age, disease grade, and smoking history. All patients were naïve, without history of hormonal therapy, radiotherapy, or chemotherapy.

### 2.2 ImageStreamX-based detection of histones and histone complexes in plasma of healthy individuals and cancer patients

Levels of histones and histone complexes in plasma were measured according to our previously described ImageStream-based multichannel detection approach [18].

### 2.3 Nucleosome detection by ELISA

To detect plasma levels of histones H3.1 and H3.3 in healthy individuals and GB patients, we used the Human Histone H3.1 (HIST1H3A) ELISA Kit (CSB-EL010418HU, Cusabio) and the Human Histone H3.3 (H3F3A) ELISA Kit (abx387728, Abbexa), respectively, according to the manufacturer's instructions. The assay was performed on undiluted samples, and the experiment was performed in duplicate.

### 2.4 Statistical analyses

Statistical analyses on the abundance of histones and histone complexes were conducted in *RStudio (version 4.4.3)*. Detection of histones H2B, H3, and H4 occurs in all three sets of antibodies. To account for the potential batch effect, the measured abundance of all histones and histone complexes was averaged and log-transformed. Pairwise Kruskal-Wallis test or Pairwise Wilcoxon rank-sum test was applied to compare the levels of plasma histones and histone complexes between groups, followed by a Dunn post-hoc test, when required. The Bonferroni method was applied to correct for multiple testing. Nonparametric Spearman correlation was applied to correlate the levels of histone and histone complexes with specific clinical parameters. Patients with missing data were excluded from the respective analyses. Boxplots and scatter plots were created using RStudio (version 4.4.3).

## 3 Results

### 3.1 Plasma levels of H3.1- and H3.3- nucleosomes in GB patients

**Fig. 1:**
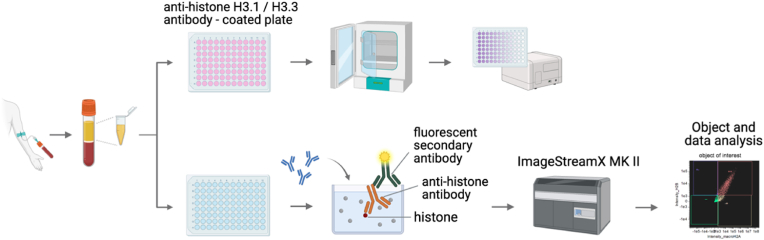
Workflow of assays used in the histone signature assessments of plasma samples from healthy people and glioblastoma patients. Plasma level detection of histones H3.1 or H3.3 in plasma by ELISA (upper row), and individual histones and histone complexes by imaging flow cytometry-adapted method (lower row).

**Fig. 2:**
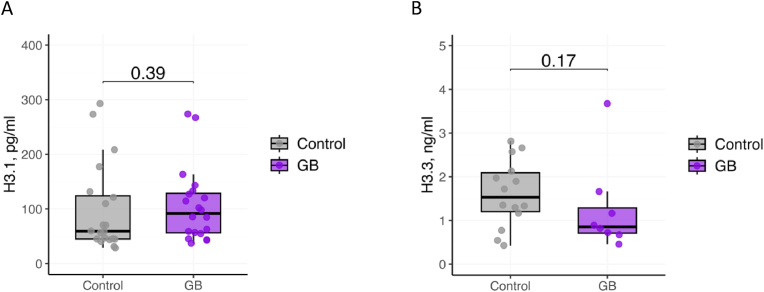
Plasma levels of **A**. histone H3.1 and **B**. histone H3.3 in glioblastoma (GB) patients, compared to controls. **A**. GB, n = 18, controls = 20. **B**. GB, n = 20, controls = 20. **A, B**. Each point represents an individual patient sample. Statistical significance is indicated by p-values on each plot.

The median age was comparable between the control (n = 30) [61.5 (54.25 - 71.25, IQ1-IQ3)] and the GB (n = 44) [62.5 (52 - 66.25, IQ1-IQ3)] patient groups (Table 1). The ratio of males/females in the two groups are 60-40% in the control cohort and 50-50% in the GB group (Table 1). 4/44 GB patients had grade 3 of the disease, while 40/44 displayed the most aggressive and advanced grade 4 [20]. A large percentage of smokers were present both in the control and in the GB groups, with no significant differences (Table 1). As mentioned above, histone H3.1 and H3.3 subtypes differ for their replication-coupled and replication-independent deposition on newly synthesized DNA, respectively, providing crucial functional differences [15]. Therefore, we measured plasma levels of histones H3.1 and H3.3 in the plasma of healthy individuals and adult GB patients using commercially available H3.1 (healthy, n = 20; glioblastoma, n = 20) and H3.3 (healthy, n = 18; glioblastoma, n = 20) Enzyme-Linked Immunosorbent Assays (ELISA) (Fig. 1, upper panel). H3.3 levels were below the threshold of detection using ELISA in 8 GB patients. On average, GB patients exhibited no significant differences in the levels of either H3.1 or H3.3 histones compared to healthy controls (Fig. 2A and B).

**Table 1:** Characteristics of study participants: control, glioblastoma (GB) patients. P-values are calculated by: Kruskal-Wallis test (for age) and Chi-square test for sex and smoking history.

*Parameters*	Controls, N = 30	Glioblastoma, N = 44	p-value
*Sex (M/F)*	18/12	22/22	0.629
*Age (years)* *Median (IQ1-IQ3)*	61.5 (54.25 – 71.25)	62.5 (52 - 66.25)	0.337
*Grade (3/4)*	NA	4/40	NA
*Smoking (Yes/No/Not reported)*	19/11	13/16/15	0.577

### 3.2 Plasma histone signature of GB patients

**Fig. 3:**
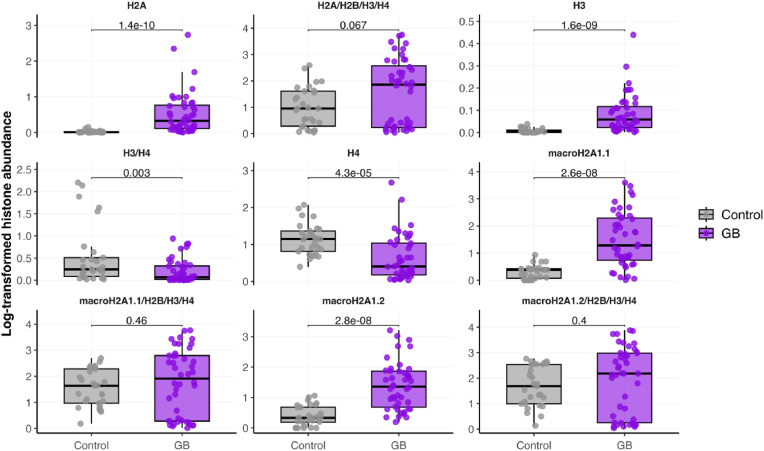
Levels of circulating histones and histone complexes in glioblastoma (GB) patients (n = 44), compared to controls (n = 30). Each point represents an individual patient sample. Statistical significance is indicated by adjusted p-values.

Next, we sought to determine if, conversely, the diversity of circulating histone complexes might be used as new biomarkers for GB. In fact, ELISA assays can detect nucleosomes or individual histones in the blood; however, real-time high-throughput detection of multiple histones remains unexplored for GB. To better characterize the histone profile in this disease, we analyzed circulating histones at multiple levels: individual histones, dimers, and tetramers/nucleosomes using our established imaging flow cytometry pipeline [17,18,[21], [22], [23], [24], [25]] (Fig. 1, lower panel). We focused on the canonical histones (H2A, H2B, H3, and H4) and the two isoforms of the macroH2A1 variant (macroH2A1.1 and macroH2A1.2). Compared to controls, GB patients showed elevated levels of individual histones H2A, H3, macroH2A1.1, and macroH2A1.2 (Fig. 3). In contrast, H4 levels were markedly decreased in glioblastoma patients. When examining nucleosome complexes (H2A/H2B/H3/H4, macroH2A1.1/H2B/H3/H4, and macroH2A1.2/H2B/H3/H4) we found comparable levels between GB and control cohorts (Fig. 3). When stratifying patients by GB grade (grade 3 vs. grade 4), we observed that, except for H2A, grade 3 patients consistently showed higher levels of histones and histone complexes than grade 4 patients (Fig. S1). These findings suggest a specific histone signature associated with GB that may serve as a potential biomarker for disease detection and classification.

**Fig. 4:**
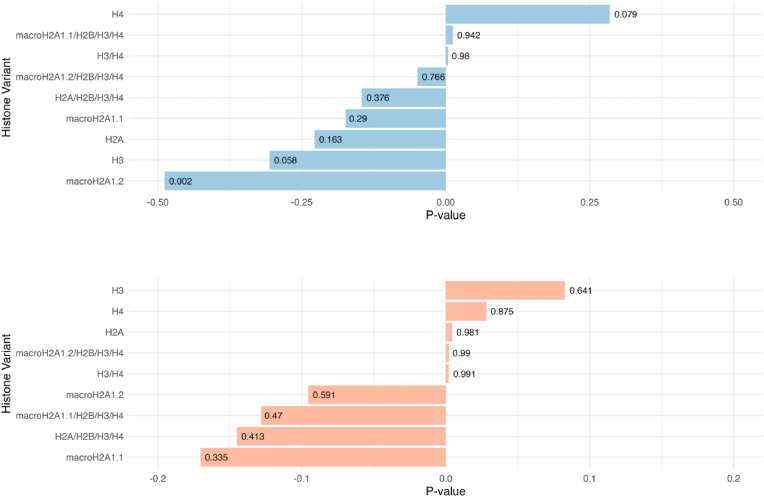
Correlation of circulating histones and histone complexes with age in **A**. male and **B**. female patients recruited in the study. Spearman's correlation was used. The direction of the correlation is shown on the graph as positive or negative. The strength of the correlation is indicated by statistical significance expressed by p-values.

**Fig. 5:**
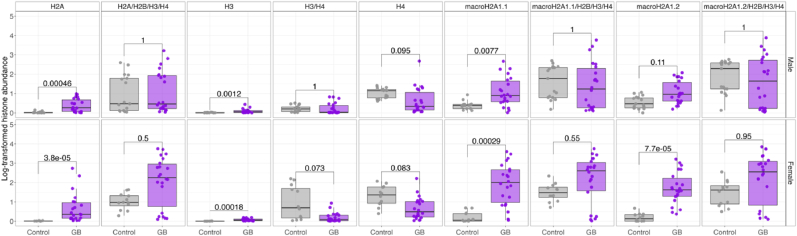
Levels of circulating histones and histone complexes in glioblastoma (GB) patients, compared to controls in males (upper panel) and females (lower panel). GB: males, n = 22; females, n = 22. Controls: males, n = 18; females, n = 12. Each point represents an individual patient sample. Statistical significance is indicated by p-values on each plot. Statistical significance is indicated by adjusted p-values.

We next assessed potential associations between plasma histone levels, demographics, and clinical parameters within the GB cohort. Age and male gender are established risk factors for developing GB [26,27]. A moderate negative correlation was observed between macroH2A1.2 levels and patient age, specifically in men (Fig. 4A and B). When stratifying the circulating histone results (Fig. 3) according to gender, histone species abundance levels were comparable in men and women (Fig. 5). Recent meta-analyses and large population studies reported conflicting or inconclusive results about the association between smoking and increased risk of GB [[28], [29], [30]]. Accordingly, no significant differences in histone profiles were found between smokers and non-smokers in our GB cohort (Fig. S2).

## 4 Discussion

In the present study, we investigated the circulating histone profile in plasma from adult GB patients compared to healthy controls using advanced imaging flow cytometry and ELISA-based approaches. Our key findings reveal a distinct histone signature in GB patients, characterized by elevated levels of individual histone monomers H2A, H3, macroH2A1.1, and macroH2A1.2, alongside markedly reduced H4 levels in GB patients. In contrast, nucleosome complexes with core H2A histone protein or macroH2A1 variants showed no significant differences between groups, suggesting that the diagnostic potential lies primarily in the dysregulation of free histone monomers rather than assembled nucleosomes. Similarly, histones H3.1 and H3.3 were detected at similar levels in glioblastoma and control plasma samples. While these results could indicate a lack of specificity, the low sensitivity of the applied ELISA approach might have also affected the findings. Additionally, stratification by tumor grade indicated higher histone and complex levels in grade 3 compared to grade 4 tumors (except for H2A and H3), hinting at potential grade-specific variations. These observations provide proof of concept for histone monomers as novel minimally invasive biomarkers in adult GB, complementing traditional diagnostic modalities [31].

A notable limitation of this study is the relatively modest sample size (n = 44 GB patients and n = 30 controls), which may constrain the statistical power to detect subtle associations or subgroup differences [32]. Although commercial plasma samples ensured consistency in collection and processing, future validation in larger, prospective cohorts with longitudinal sampling is warranted to confirm the robustness and clinical utility of these histone signatures. Pediatric and adult GB exhibit profound biological and diagnostic differences, underscoring their classification as distinct entities in the 2021 WHO guidelines [33]. Adult GB predominantly arises in cerebral hemispheres, frequently harbors IDH-wildtype status with EGFR amplification, PTEN loss, and TERT promoter mutations, and follows aggressive primary or secondary pathway [33,34]. By contrast, pediatric high-grade gliomas, including GB, often involve midline structures (e.g., diffuse midline glioma/DIPG with H3K27-altered status), display recurrent histone H3 mutations (K27M in H3F3A or HIST1H3B), and show distinct metabolic profiles, such as higher glucose utilization and lower lactate production [33,35]. These molecular divergences contribute to varying prognoses and therapeutic responses, with pediatric cases generally rarer but potentially more amenable to certain targeted approaches [34,36].

Our previous work on pediatric H3K27-altered diffuse midline glioma (DMG/DIPG) [18] highlighted a contrasting circulating histone profile: significant upregulation of dimers and tetramers (e.g., macroH2A1.1/H2B, macroH2A1.2/H2B, H2A/H2B, H3/H4, and full nucleosomes) coupled with downregulation of individual monomers (H2B, H3, H4) [18]. This pattern, observed in plasma, CSF, and tumor cell supernatants, suggests enhanced nucleosome release or stability in pediatric DMG/DIPG, potentially reflecting greater tumor necrosis or distinct epigenetic dysregulation driven by H3K27M mutations [37,38]. The inverse profile in adult GB (elevated monomers and reduced H4) indicates differential mechanisms of histone release, processing, or clearance, aligning with age-specific oncogenic drivers and tumor microenvironments [39,40]. These findings reinforce the need for age-tailored liquid biopsy strategies, as adult GB histone signatures may better capture monomer dysregulation rather than complex assembly [41]. We observed moderate correlations between histone levels and clinical parameters in the GB cohort. Notably, macroH2A1.2 showed a negative association with age and higher levels in females compared to males. Established GB risk factors include older age (peak incidence 65-75 years) and male predominance (male:female ratio ∼1.6:1), with males often exhibiting worse outcomes [42,43]. The age correlation may reflect cumulative epigenetic alterations or reduced immune surveillance in older patients, while gender differences could stem from hormonal influences (e.g., estrogen-mediated chromatin regulation) or X-chromosome-linked factors affecting macroH2A variants [44,45].

Circulating histones are predominantly released from hematopoietic cells during processes such as NETosis, apoptosis, or immune activation, rather than solely from tumor necrosis [46,47]. Given the strong interconnections between GB and hematopoiesis, evidenced by tumor infiltration with hematopoietic stem/progenitor cells (HSPCs), myeloid-derived suppressor cells, and bone marrow-derived macrophages that promote immunosuppression and progression [[48], [49], [50]], we hypothesize that elevated histone monomers in adult GB plasma may originate primarily from the hematopoietic compartment. Tumor-driven signals such as G-CSF/GM-CSF may skew bone marrow hematopoiesis toward myeloid lineages [51,52], potentially enhancing the release of extracellular histones from immature or activated myeloid cells within the tumor microenvironment or systemically.

A particularly relevant mechanism linking hematopoiesis to circulating histones involves the formation of neutrophil extracellular traps (NETs), a specialized form of innate immune cell death where neutrophils release extracellular chromatin structures composed of DNA, histones, and granular proteins [53]. NET formation has been implicated in tumor progression, immune modulation, and metastasis, and can be induced by both tumor-derived inflammatory signals and anticancer therapies [54]. These observations support the hypothesis where GB-associated systemic inflammation promotes myeloid activation and NETosis, thereby contributing to elevated circulating histone monomers. Nevertheless, the origin of circulating histones in GB is likely multifactorial. Tumor cell death represents an additional major source, particularly given that GB is characterized by extensive necrosis and hypoxia-driven apoptosis [55]. While direct secretion of histones by tumor cells cannot be excluded, the predominance of histone monomers over nucleosomal complexes observed in our study may favor an immune-derived origin. In this context, NETosis can promote chromatin decondensation and enzymatic processing of histones, which may generate extracellular histones that are less tightly associated with DNA [[56], [57], [58]], although the precise structural state of circulating histones *in vivo* remains incompletely defined. Future studies aimed at testing this hypothesis could involve correlating histone levels with markers of peripheral myeloid cell subsets, HSPC mobilization, and NETs in GB patients. Moreover, studies combining epigenomic profiling on tumor tissues (including NGS-based methods such as ChIP-seq and ATAC-seq) may help link circulating histone dynamics to transcriptional programs underlying GB phenotypes [59,60]. In summary, our data establish a unique circulating histone monomer signature in adult GB, distinct from pediatric counterparts, with potential diagnostic implications. Addressing limitations through larger studies and mechanistic explorations of hematopoietic origins could advance histone-based liquid biopsies toward clinical translation, offering noninvasive insights into GB biology and monitoring.

## Informed consent

All participants have signed an informed consent form before their enrolment in the study.

## Ethics

The study was approved by the Institutional Ethics Committee of MU-Varna with a protocol №115/31.03.2022.

## Authorship

MV designed the study; DKT, DB performed the research, SM, YE contributed important reagents; DKT and DB collected data; DKT analyzed data; JF, YE, ABT, GC and JC provided supervision and intellectual input; DT and MV wrote the paper. All authors have reviewed and given consent to this submission of this manuscript.

## Funding

This work was supported by the Ministry of Education and Science of Bulgaria under the National Scientific Programme “Excellent Research and People for the Development of European Science” 2021 (VIHREN) of the Bulgarian National Science Fund, contract #KP-06-DV/4 from 15.12.2021. The funder had no role in study design, data collection and analysis, decision to publish, or preparation of the manuscript.

## Declaration of competing interest

The authors declare that they have no known competing financial interests or personal relationships that could have appeared to influence the work reported in this paper.

## Data Availability

The data that support the findings of this study are available from the corresponding author upon reasonable request.
