# Supplementary material for: Plasma histone monomers as novel diagnostic markers in adult glioblastoma
Source: J Liq Biopsy. 2026 Apr 5;12:100464. doi: 10.1016/j.jlb.2026.100464 (PMC13090717; doi:10.1016/j.jlb.2026.100464)
Supplement: Multimedia component 1 [file mmc1.docx]

**
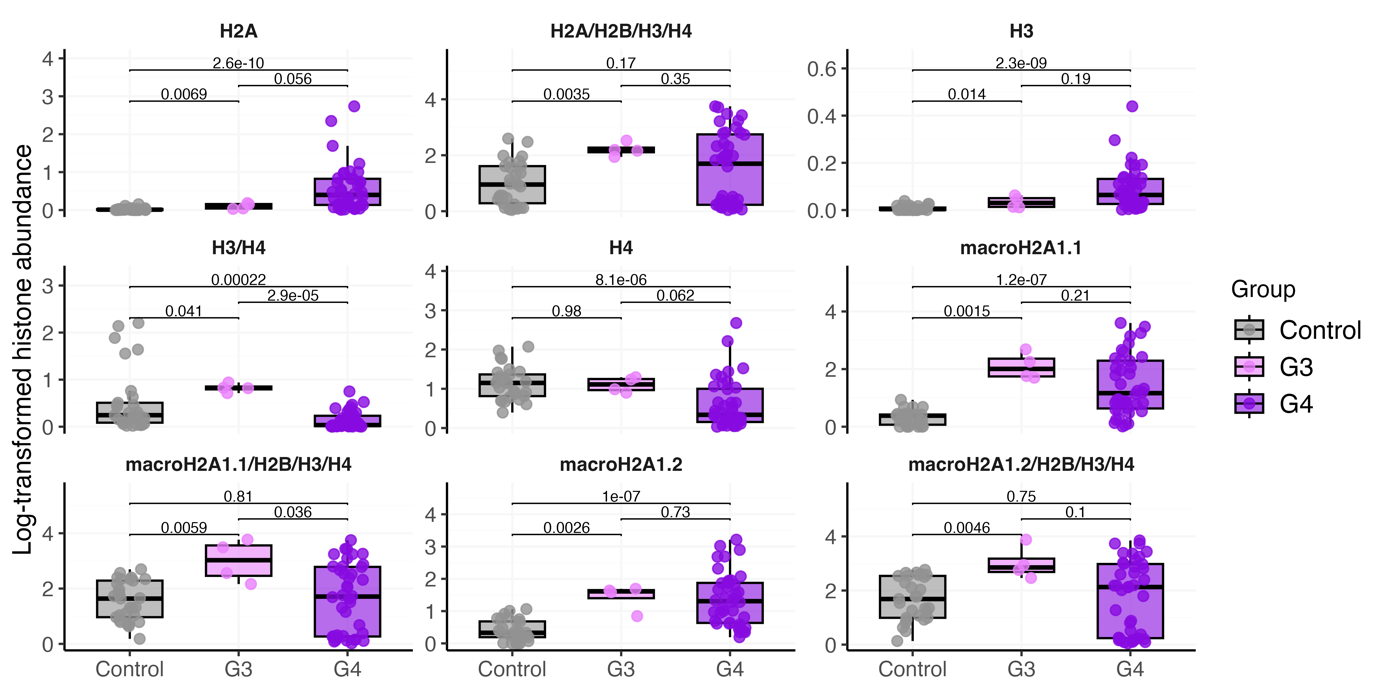
**

**Supplementary Figure 1**. Comparison between the plasma levels of circulating histones and histone complexes in glioblastoma (GB) patients, stratified by grade and compared to controls. Grade 3 (G3), n=4; grade 4 (G4), n=40; controls, n=30. Each point represents an individual patient sample. Statistical significance is indicated by p-values on each plot. Statistical significance is indicated by adjusted p-values.


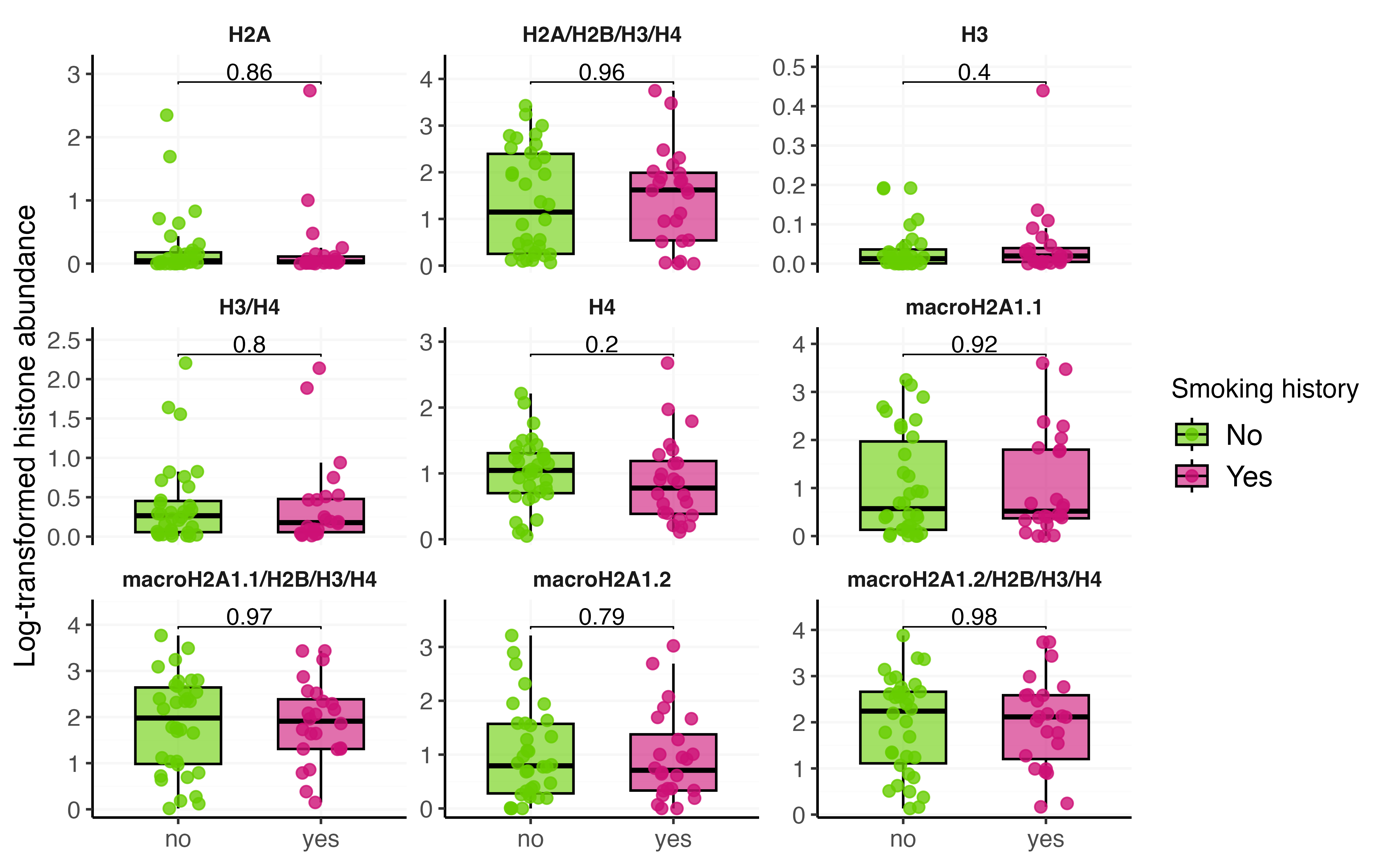


**Supplementary figure 2**. Comparison between the plasma levels of circulating histones and histone complexes in glioblastoma (GB) patients and controls, subgrouped by smoking history status. Current and past smokers are considered with smoking history (GB, n=13; controls, n=19). Patients that have indicated no current or past smoking are considered as non-smokers (GB, n=15; controls, n=11). Statistical significance is indicated by adjusted p-values.
